# Full Ground Ultra-Wideband Wearable Textile Antenna for Breast Cancer and Wireless Body Area Network Applications

**DOI:** 10.3390/mi12030322

**Published:** 2021-03-19

**Authors:** Sarmad Nozad Mahmood, Asnor Juraiza Ishak, Tale Saeidi, Azura Che Soh, Ali Jalal, Muhammad Ali Imran, Qammer H. Abbasi

**Affiliations:** 1 Department of Electrical and Electronic Engineering, Faculty of Engineering, Universiti Putra Malaysia, Serdang 43400, Malaysia; azuracs@upm.edu.my; 2 Electrical and Electronic Engineering Department, Universiti Teknologi PETRONAS, Bandar Seri Iskandar 32610, Malaysia; tale_g03470@utp.edu.my; 3 College of Information Engineering, Al-Nahrain University, Al-Jadriya Complex, Baghdad 10070, Iraq; ali.sadeq@coie-nahrain.edu.iq; 4 Communications Sensing and Imaging Group, James Watt School of Engineering, University of Glasgow, Scotland G12 8QQ, UK; muhammad.imran@glasgow.ac.uk (M.A.I.); Qammer.Abbasi@glasgow.ac.uk (Q.H.A.); 5 Artificial Intelligence Research Centre (AIRC), University of Ajman, Ajman 346, United Arab Emirates

**Keywords:** full ground antenna, UWB antenna, high gain antenna, breast cancer, microwave imaging

## Abstract

Wireless body area network (WBAN) applications have broad utility in monitoring patient health and transmitting the data wirelessly. WBAN can greatly benefit from wearable antennas. Wearable antennas provide comfort and continuity of the monitoring of the patient. Therefore, they must be comfortable, flexible, and operate without excessive degradation near the body. Most wearable antennas use a truncated ground, which increases specific absorption rate (SAR) undesirably. A full ground ultra-wideband (UWB) antenna is proposed and utilized here to attain a broad bandwidth while keeping SAR in the acceptable range based on both 1 g and 10 g standards. It is designed on a denim substrate with a dielectric constant of 1.4 and thickness of 0.7 mm alongside the ShieldIt conductive textile. The antenna is fed using a ground coplanar waveguide (GCPW) through a substrate-integrated waveguide (SIW) transition. This transition creates a perfect match while reducing SAR. In addition, the proposed antenna has a bandwidth (BW) of 7–28 GHz, maximum directive gain of 10.5 dBi and maximum radiation efficiency of 96%, with small dimensions of 60 × 50 × 0.7 mm^3^. The good antenna’s performance while it is placed on the breast shows that it is a good candidate for both breast cancer imaging and WBAN.

## 1. Introduction

In this paper, we introduce an ultra-wideband (UWB) antenna that can be utilized in two major applications: (1) wireless body area network (WBAN) and (2) breast cancer im-aging. A wireless body area network (WBAN) is a collection of low-power, miniaturized, lightweight wireless sensor nodes that monitor human body functions as well as the surrounding environment. WBANs play a very important role in the field of health services, enabling personal data monitoring, and are a leading area of research for health and disease management as well as monitoring human physiological activity such as health status [1]. The lightweight, ultra-low power wearable sensors in a WBAN can be classified as off-body, on-body, or in-body. These sensors can communicate wirelessly using both electromagnetic coupling and RF (radio frequency) communications. Wearable technologies have been used to monitor various parameters of the human body [2]. Wearable antennas as a vital part of WBAN systems can be used to send and receive pulses to the human body like a wearable bra to check a woman breast for the risk of tumour existence. Using this wearable bra as a part of a WBAN system, the patient will not be needed to go to the hospital, especially in the situation of COVID-19. Another application of UWB antennas is in the microwave imaging of breast cancer. The imaging is an alternative method to X-ray mammography, which has the advantage of no harmful radiation, no pressurized imaging that provides the ease of more frequent imaging, which can be a key in the early detection of tumours.

There are multiple challenges in designing antennas in the presence of the human body, including the effects of high loss and high permittivity tissues [3]. All wearable antennas operate in close proximity to the high dielectric medium of the human body or have close interaction with the human body that affects the radiation characteristics of the design. As relative permittivity increases near the human body, it affects the Q factor of the wearable design and electric flux is suppressed [4]. Antenna performance therefore degrades when the antenna touches the body. Thus, antennas must be designed carefully to maintain performance when they touch a medium other than free space [5]. Furthermore, wearable designs must comply with standards for specific absorption rates (SARs). Several planar structures, metamaterial (MTM) structures [6], ferrite sheets [7], soft surfaces, frequency selective surfaces (FSS), and large ground planes [8] have been used in body area network (BAN) applications as insulating layers to protect the human body from unwanted radiation [9,10,11].

Bending and stretching of flexible and wearable antennas during use not only affects resonance frequency but also the radiation characteristics of the wearable antenna, especially when it requires circular polarization. Wearable antennas will inherently face different crumpling and bending conditions [12,13,14]. Human movements result in unpredictable, asymmetrical crumpling, hence, it is unreasonable to study a wearable antenna in a symmetrical crumple case [15]. Besides, controlling all these effects and keep consistency in performance during different bending conditions was performed to ensure that the resonant frequency operates within the required region [16] for multi-band or dual-band application. Flexible antennas (transparent and non-transparent) require high mechanical robustness and a high degree of bending (up to 90°) [17]. Antennas intended for on-body use should therefore be designed in such a way that radiation characteristics are not changed dramatically by bending [6,17,18]. When the antenna dimensions are small, the antenna can be more robust and less affected by bending [11,19,20]. Miniaturization has been a key factor in improving the performance of wearable antennas.

Several shapes have been suggested for wearable antennas, like planar Inverted-F antennas (PIFA) [21,22], magneto-electric dipole antennas [23], substrate-integrated waveguide (SIW) antennas [24,25], electromagnetic band gap (EBG)-based antennas [26], dipole antennas [27], fractal-based patch antennas, circularly polarized integrated filtering (CPIF) antennas, surface wave parasitic array (SWPA) antennas, and cavity slot monopoles [28]. In addition, Yagi-Uda antennas were proposed as candidates for on-body millimetre-wave communications, representing a good prototype in terms of size and gain performance [29]. Theses antennas should be stable electrically and mechanically robust to not to get affected by movement [17]. Considering these requirements, several problems occur during the fabrication of wearable antennas [30,31,32,33,34].

Few wearable ultra-wideband (UWB) antennas have been designed specifically for use in breast cancer imaging. One of the most relevant wideband antennas was designed on a flexible polyimide substrate (εr=3.5−3.8) and obtained bandwidth (BW) of 2–5 GHz with dimensions of 20 mm × 20 mm. Then, it was embedded in a bra to detect a tumour in the breast [35,36]. Due to its narrow bandwidth, the system could not detect the tumour properly and with precision, and several clutters that appeared around tumour. Spatial resolution and range resolution (ρR) (inversely proportional to the effective bandwidth of the pulse) are two factors that indicate whether a specific antenna design and imaging system can detect tumours and show the minimum dimension of the tumours they can detect. In addition to those, some flexible wearable antennas are designed for 5G and IoT applications [37,38,39,40,41].

The UWB antennas presented in the literature, both wearable and non-flexible, showed limitations on the resolution, high SAR, and low performances having larger dimensions. A wearable antenna intended for use in detecting breast tumours should have broad BW, low SAR, and small dimensions to detect tumours effectively [35,36].

## 2. Antenna Configuration

**Figure 1:**
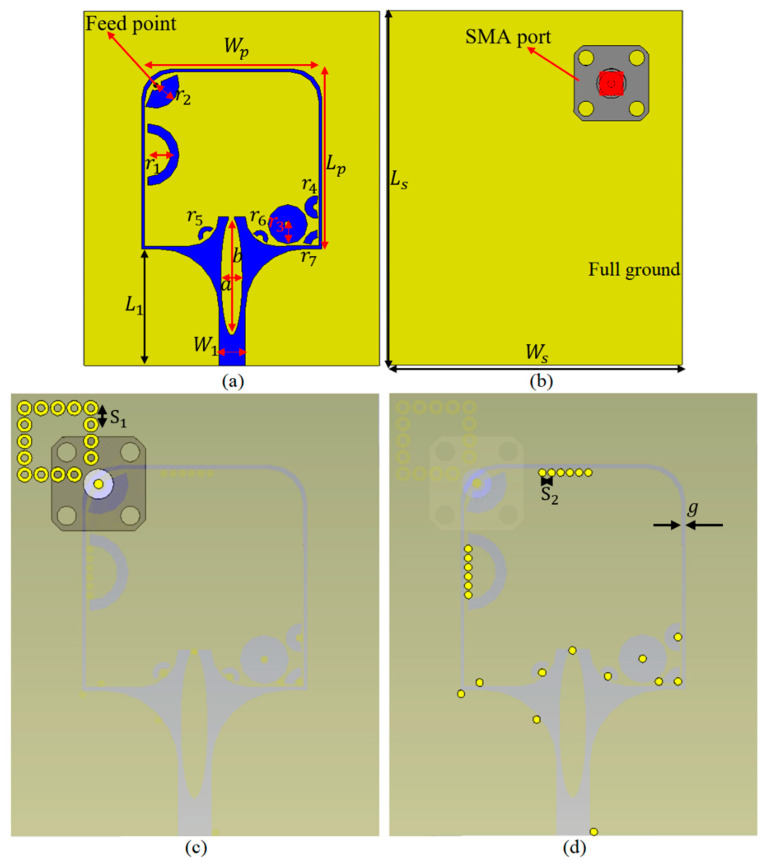
(**a**,**b**) Simulated prototype of the proposed antenna: (**a**) front view and (**b**) back view and the full ground, (**c**) substrate-integrated waveguide (SIW) feeding transition, and (**d**) loading with shorting pins and photonic band gap (PBG) structure.

**Figure 2:**
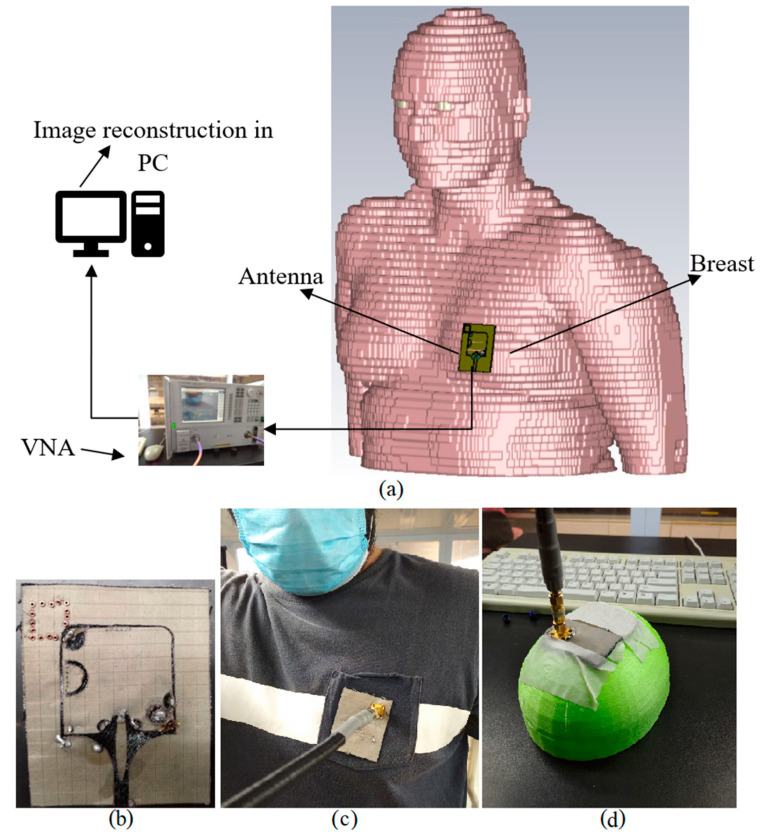
(**a**) Simulated human female voxel model and simulation setup, (**b**) the fabricated prototype of the antenna, (**c**) measurement setup on chest, and (**d**) the measurement setup on breast phantom.

To design a flexible, miniaturized, wearable UWB antenna, a substrate with a high dielectric constant and low loss tangent can be utilized (the dielectric constant of the flexible antenna’s substrate should be close to the breast’s relative permittivity to have excellent coupling between the antenna and the breast [3,42]. This improves the scattering parameters of the antenna). The proposed antenna design is arrived at after designing the conventional patch and enhancing gain and BW using techniques such as photonic band gap (PBG) structures and substrate integrated waveguide (SIW) (Figure 1). In all steps of designing the antenna, the dimensions and patch are optimized to obtain the best results. Figure 2 and Table 1 show the simulation and measurement setup.

Figure 2a depicts the general concept of the simulation setup of the proposed antenna. It demonstrates that the antenna touches the breast and then the scattering data are recorded by a vector network analyser (VNA). Afterward, the data extracted from VNA and imported to a PC to reconstruct the image of a tumour in breast using an algorithm. Figure 2b,c indicates the front view of the fabricated prototype of the antenna and the measurement setup of the antenna on chest, respectively. It should be mentioned that the antenna is pasted on the shirt not to move during the measurement. Figure 2d shows the measurement setup of the antenna located on a breast phantom. This phantom is 3D printed using elastic PLA material considering almost the size of a *E* size female breast.

**Figure 3:**
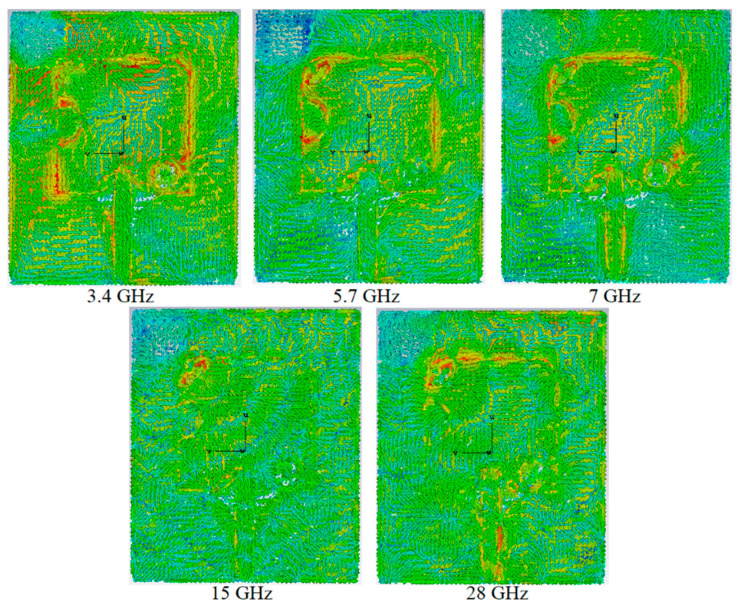
The surface current distribution (SCD) at 5G, sub-6 GHz, lower-end, and higher-end of the working band.

During the antenna optimization, size alteration of each design parameter in the antenna design procedure affects the antenna characteristics such as surface current distribution (SCD) shown in Figure 3. This happens because each change alters the surface current distribution of the antenna and so as the electromagnetic fields around it. Analysing these behaviours illustrates how each variation in these parameters affects the antenna characteristics. Therefore, the SCD is investigated at different frequencies such as 3.4 GHz (5G), 5.7 GHz (sub-6 GHz), 7 GHz (lower-end of the BW), 15 GHz, and 28 GHz (higher-end of the BW). It shows that the current is stronger around the feeding and the slot that separates the patch from the CPW slot at 3.4 GHz. it is noticed that this current is stronger around the EBG structures and the arc shape slots, which were added to extend the BW at the lower band (7 GHz) and make the stopbands occurred around 12.5 and 16.5 GHz to passbands. Besides, it depicts that the current is stronger and has more density around the CPW slot and the feeding at the lower-end and higher-end of the operating BW.

There has been in-depth study of various periodic structures in microstrip lines, including photonic bandgap (PBG), electromagnetic band gap (EBG), and defected ground structure (DGS) [43]. These three periodic structures each have their own properties and advantages. EBG structures can be considered a periodic dielectric or metallic materials, which have the capability of passing or stopping the propagation of electromagnetic waves at a known frequency. PBG materials can be applied to increase the behaviour of a single element patch antenna to obtain a higher gain. EBG, like PBG, structures can be also exploited to make a low-profile and high-efficiency antenna. Moreover, their high surface impedance cooperates to suppress the surface waves.

EBG can be etched from the ground for added as load for the antenna to improve the performance by creating passbands in the working BW of the multiband antennas [44]. An important capability of EBG structures is their ability to decrease back radiation and the SAR (specific absorption rate). The SAR shows the rate of absorption of RF energy in the body; according to FCC guidelines, it should be less than 2 W/kg.

Designing of wideband and UWB antennas usually use conventional monopole with a ground length of λ/8. Using a truncated ground in designing a wearable UWB antenna significantly increases the SAR of the antenna, which affects the antenna’s performance and has negative, unacceptable impacts on the body. Therefore, the ground should be complete to reduce the SAR value. However, utilizing a full ground utterly disturbs the wideband working of the antenna, making it a narrowband antenna. For further improvement, the SIW structure is used to both improve the impedance matching of the antenna and degrade the SAR level.

## 3. Proposed Antenna for On- and Off-Body Conditions

As aforementioned, one of the techniques that degrade the SAR values and its negative effects on body is using a full ground in the antenna structure during the design procedure. However, using a full ground makes the working bandwidth work as a narrowband, since the ground length is much greater than λ/8 mm. Therefore, the antenna structure should be improved in a way to widen the BW. To design the antenna, a conventional rectangular patch using the equations presented in [14,20] is designed. The proposed antenna is fed by a coplanar waveguide (CPW)-fed rectangular slot and SIW transition. Then it is fed from behind through a SubMiniature version A (SMA) port and coaxial cable as depicted in Figure 1.

The proposed antenna is the first design using the equation presented in [14,20] and using the transmission technique. The feeding technique is changed to CPW and feeding is done from behind due to the concept of full ground. Feeding from behind is more profitable when using a full ground and wideband or UWB. The conventional rectangular slot uses CPW-fed feeding from behind. In addition, the materials used to design the antenna are flexible textile materials: denim (with a 1.7 dielectric constant and thickness of 0.7 mm) as substrate and ShieldIt conducting (0.17 mm) flexible material as the conductor and/or resonator.

After obtaining results from the conventional antenna, some stopbands were noticed in the working BW, especially at the higher band above the centre frequency of the antenna. Therefore, the rectangular patch and the CPW ground’s edges were curved to reduce the undesired surface waves and improve the stopbands at bands above 15 GHz and convert them to passbands. It also widens the BW at higher bands. The edge and the angle of chamfering are optimized in each step to obtain the best results.

After checking the results obtained from chamfering the edges of the CPW ground and the rectangular patch, the antenna transition feeding from the ground coplanar waveguide (GCPW) to the SIW is started. This new transition using SIW for the feeding broadens the BW and the impedance matching results of the antenna at the lower bands below the centre frequency. Furthermore, the rectangular slot and latter arc slot act as a coupling slot, which leads to a smooth transition (Figure 1c) [45,46].

The higher band above 15 GHz is achieved well and is within the working BW. However, the lower band below the centre frequency has yet to be reached. Two sets of shorting pins are located as EBGs to shift the whole BW to the lower band and increase the BW towards the lower band. In addition, these EBG pins are driving the current to the patch and distributing it to all parts of the antenna (Figure 1d).

After expanding and widening the BW and meeting the UWB requirements, the remaining stopbands can be removed by loading the antenna with shorting pins and arc slots cut from each pin based on the surface current distribution at each associated frequency. These shorting pins are located at locations where the electric field is zero or surface current distribution is high. Thus, the electric field and surface current distributions should be considered in order to convert the stopband to a passband at each desired frequency. Moreover, the capacitive and conductive loading of the antenna using the sorting pins changes the flow of the surface current to increase the radiation efficiency of the antenna. In addition, utilizing the shorting pins and arc slots produces additional resonances at 3.8 and 5.7 GHz for both ISM and 5G applications, respectively.

**Figure 4:**
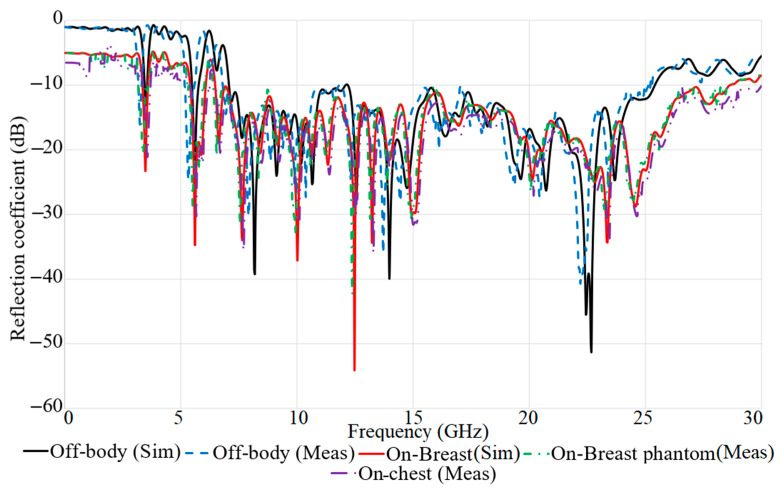
The reflection coefficient result of the proposed antenna on-body (on breast) and off-body for simulation (sim) and measurement (meas).

Figure 4 depicts the reflection coefficient result of the proposed antenna for both on-body and off-body conditions. Off-body means in free space. The off-body condition should be considered to see how much the results change between when the antenna is in free space and when it encounters the body. Air’s dielectric constant is one, which is significantly different than those of breast, skin, and tumour tissue. Hence, the antenna must be designed and optimized so that the reflection coefficient, radiation pattern, fidelity, and other parameters mentioned above do not change dramatically with placement. Figure 4 shows that the working BW for on-body conditions does not deviate from the off-body conditions dramatically. However, the working BW is reduced slightly when the antenna touches the body. All the resonances are obtained and minimal shifts in resonance occurred.

**Figure 5:**
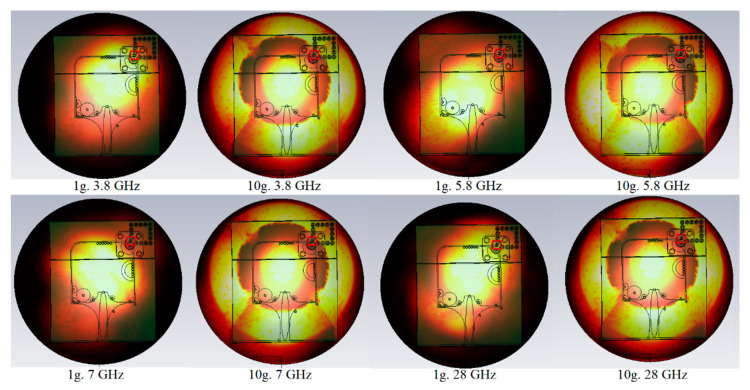
Specific absorption rate (SAR) variation on breast at different frequency and standards (1 g, 10 g).

A vital parameter that should be defined when a wearable antenna concerned is the SAR value. Industry and government standards dictate that it be less than 2 W/Kg for both standards, 1 and 10 g. Table 2 shows that the antenna offers acceptable SARs at different frequencies using both standards. In addition to Table 2, Figure 5 depicts the SAR distribution on-body (breast). To measure the SAR on the body, a layer of skin, breast fat, muscle, and bone are used; the antenna is located near the breast (4 mm distant from the breast) to take the measurement.

**Figure 6:**
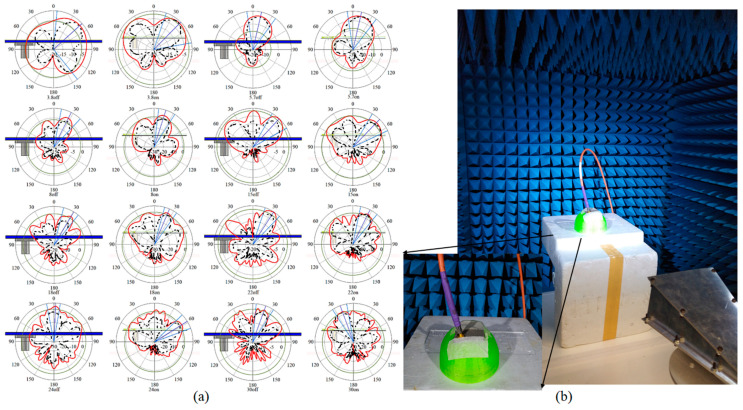
(**a**) Radiation pattern of the antenna at different frequencies on-body (breast phantom) and off-body (solid line: simulated, dashed line: measured) and (**b**) radiation measurement setup on breast phantom.

When an antenna is designed for the purpose of image reconstruction, having a stable radiation pattern is important. Figure 6 shows the radiation pattern of the antenna at the resonant frequencies and lower-end and higher-end of the working BW. In addition, it shows that the radiation pattern does not change at different frequencies by more 20°.

**Figure 7:**
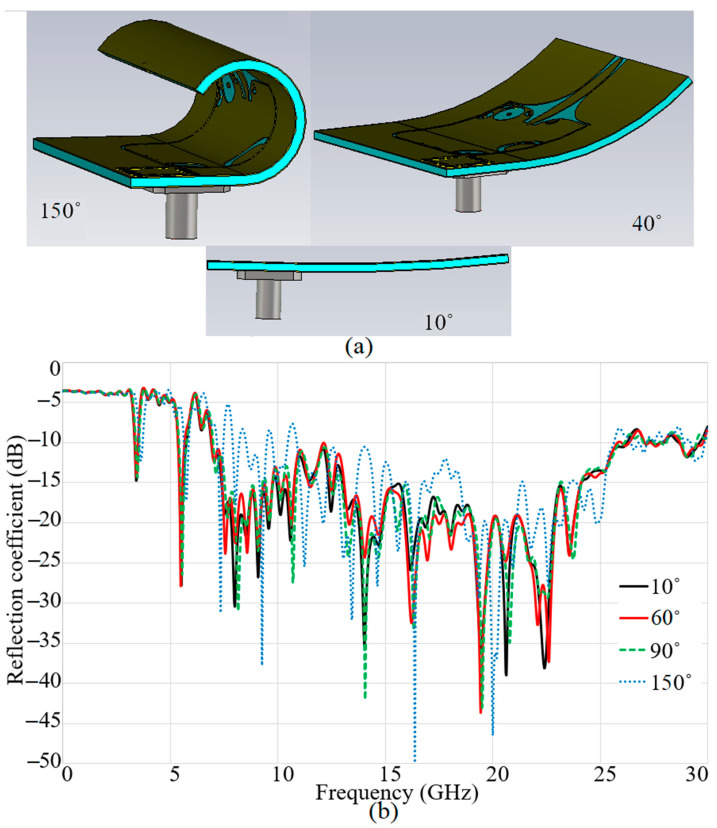
The antenna’s reflection coefficient results in different bending degrees: (**a**) bended prototype and (**b**) reflection coefficient results.

The antenna’s robustness in harsh environments and under different bending conditions must be investigated to ensure performance is not affected drastically. Besides, this investigation should be done for the conditions when the wearable antenna must be bended as it is positioned on a breast or embedded in a bra as wearable imaging device. Therefore, the antenna is bent up to 150°, as presented in Figure 7, to determine whether the reflection coefficient result is disturbed (the bending angle starts from 10° to 150° to check different angles of bending because an actual breast does not have a homogenous shape and it requires different bending angles to be considered). Figure 7 shows that the reflection coefficient results were not altered extremely by bending conditions up to 140°. Most of the BW was still attained and the other resonances at 3.8 and 5.7 GHz were obtained as well, apart from a few stopbands occurring in the BW.

**Figure 8:**
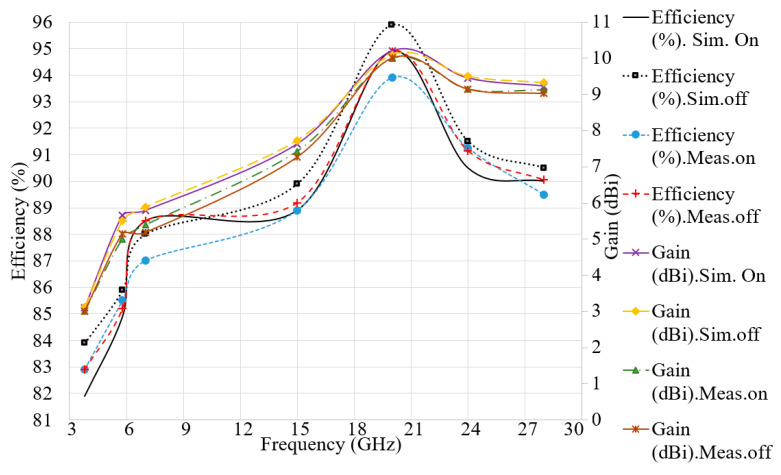
Simulated and measured gain and efficiency for both on-body (breast) and off-body (free space) conditions.

Simulated and measured results of gain and radiation efficiency for both on- and off-body conditions (on-body: on breast) are presented in Figure 8 and Table 3. It shows that the maximum gain and radiation efficiency are 10.1 dBi and 96%, respectively. In addition, radiation efficiency of more than 82% is obtained for the entire working BW. Once the antenna’s radiation characteristics have been investigated and evaluated for on- and off-body conditions, the antenna’s capability in image reconstruction of tumours should be assessed.

### Proposed Antenna’s Capability for Image Reconstruction

**Figure 9:**
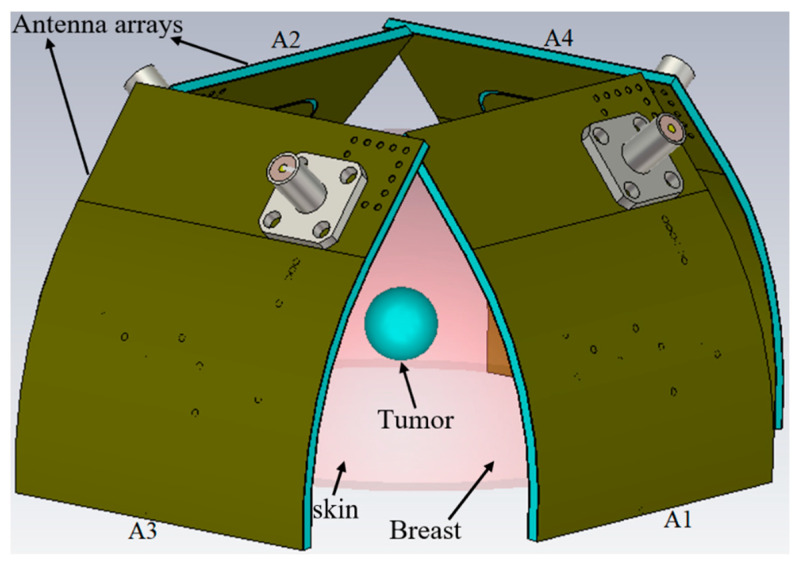
Simulated setup of the antenna array elements in on-body conditions, A1: antenna array 1; A2: antenna array 2; A3: antenna array 3; A4: antenna array 4

The operation of the proposed wearable UWB antenna will be shown in both time and frequency domains. Narrowband antennas are usually propagated and described in the frequency domain, and their radiation characteristic are considered constant over a few percent of the working bandwidth. Since the UWB devices are often known as an impulse-based technology, the antenna’s property in time-domain is to be investigated when a continuous wave illuminates the transmitter antenna [3,20]. On the other hand, a two-dimensional vector with two orthogonal polarization components is assumed in frequency domain. However, the transient response of an antenna varies principally with time, along with the angles of departures and polarization. Figure 9 shows the simulation setup and how a UWB pulse is sent and received between every two UWB antennas [20].

**Figure 10:**
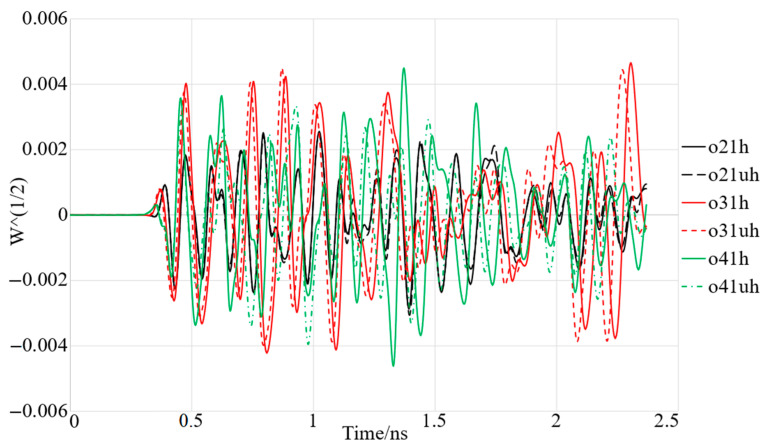
Simulated setup of the antenna array elements in on-body conditions (with (uh) and without (h) tumour).

Before starting the image construction of a tumour in the breast several vital factors affecting these processes should be investigated and evaluated. Figure 9 shows the simulation setup of four array elements of the proposed wearable antenna around the breast phantom with diameter of 100 mm. The received signals from the distinct arrays should be investigated first. The received signals and the time delay are used in the signal analysis to reconstruct the image of the tumour. The received signals from the different array elements (A2–A4) in both on- and off-body conditions are presented in Figure 10. Array one (A1) transmits and the other arrays receive the signals. It was noticed that the signals’ shape did not change when they faced the breast model, while the signal’s amplitude differed, and they shifted.

**Figure 11:**
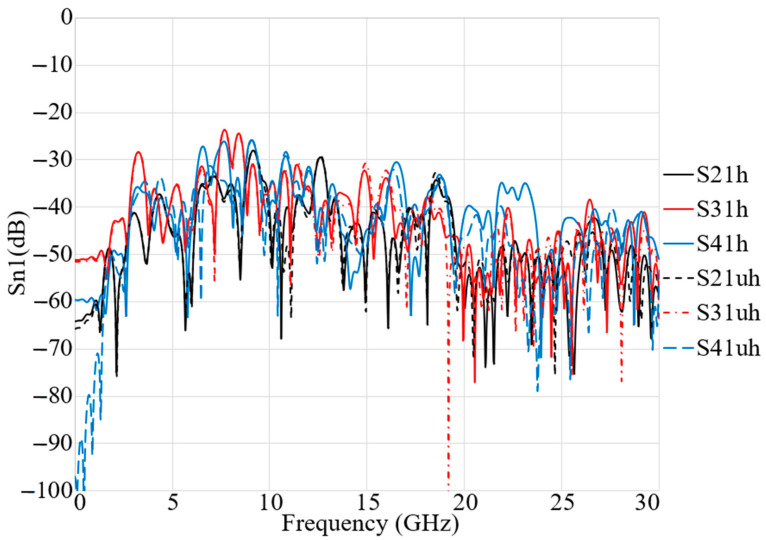
Simulated setup of the antenna array elements in on-body conditions (with (uh) and without (h) tumour).

**Figure 12:**
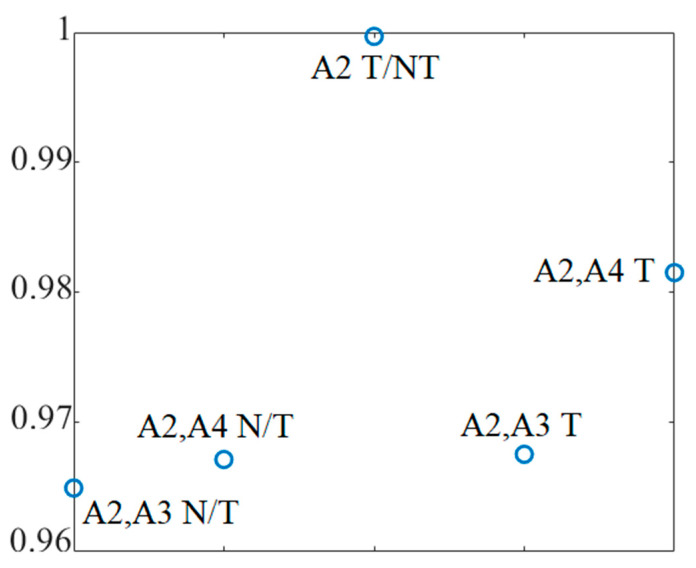
Simulated setup of the antenna array elements in on-body conditions (with tumour (T) and without tumour (N/T)).

Figure 11 shows the transmission coefficient results of the arrays arranged around the breast model as shown in Figure 9. Figure 11 illustrates that a perfect isolation and low mutual coupling is observed: the transmission coefficient level is less than −25 dB for all arrays, in situations both with and without tumours. The fidelity factor is a parameter that plays important role in imaging. It shows the similarity among the received signals from different arrays in different angles. In addition, it shows by how much a signal is distorted when it transmits from the transmitter and is received by the other arrays. Figure 12 shows high fidelity among the received signals from different arrays for on-body conditions (with and without the presence of tumours).

**Figure 13:**
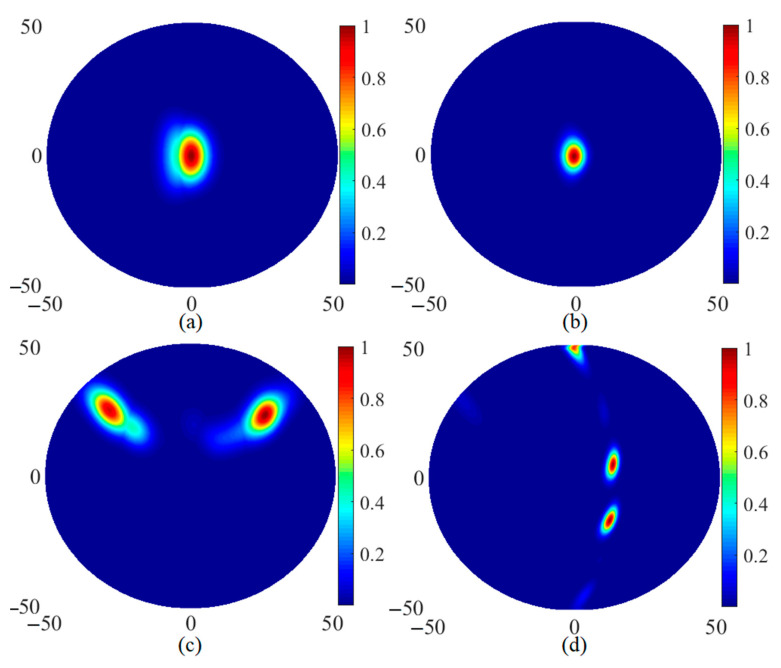
Reconstructed images using received signals from four arrays and a robust time reversal algorithm [47]: (**a**) central tumour with skin, (**b**) central tumour without skin, (**c**) two tumours and (**d**) three tumours.

After the proposed antenna’s characteristics were investigated and evaluated in both on- and off-body conditions (and with and without tumours), the antenna’s capability in image reconstruction of tumours in the breast was assessed under various conditions: with a central tumour (with and without skin) and with two and three tumours. Figure 13 shows the reconstructed image using the robust time reversal algorithm presented in [47]. The spherical tumours were perfectly detected under all four conditions. Only very small ignorable clutters were detected in these images.

After investigating and evaluating the proposed antenna’s performance under various conditions, it can be concluded that the proposed antenna is a reliable candidate as a wearable device for controlling breast cancer at its early stage.

Table 4 illustrates the close comparison of the proposed work with some recent similar works. It shows that the proposed antenna achieved wider BW, higher gain and radiation efficiency, and lower SAR values. The proposed antenna obtained these outcomes with smaller dimensions (it should be mentioned that the width of an antenna plays a direct impact on its efficiency and BW: when an antenna has larger dimensions, both gain and efficiency increase [42,48]). Among these works, only one, presented in [49], used a full ground. When a full ground is used, obtaining a wide BW is very difficult and most UWB, wideband, and broadband antennas use truncated grounds in order to increase BW.

## 4. Conclusions

The WBAN technologies have been used for vast applications such as health monitoring and surveillance. Using light, low profile, and low power consumption sensor node, they could usually examine and monitor patient health (such as patient having breast cancer) and then cast the collected data wirelessly. The wearable technologies, which utilized flexible and wearable electronic devices like antennas, usually faced challenges especially when they are integrated and placed next to the human body. They required to be comfortable and robust enough against any sudden movement of body in harsh environment. Regarding the patients having breast cancer, the conformability of the system always was an issue. Besides, at the situation of the COVID-19 taking the patients to hospital proved to be risky for the patients. Therefore, having a wearable imaging device that can monitor the possibility of existing tumour in breast at home will be helpful. One of the important parts of a wearable WBAN device used for health monitoring such as breast cancer is the antenna. This antenna should have broad BW (like UWB antennas) in order to have higher resolution and accuracy in image reconstruction of the tumour in breast.

One of the most important challenges during the design process of a wearable UWB antenna is the SAR values. However, to obtain the desired ultra-wide bandwidth, truncated grounds are primarily used in wearable antenna structures, which enhances the SAR value, and it is not desired in wearable antennas. Therefore, a UWB antenna comprising a full ground is designed, simulated, and measured to achieve a broad BW with stable radiation characteristics considering the SAR within the acceptable range according to the standards. The proposed antenna is designed on a denim substrate, with a thickness of 0.7 mm and $\varepsilon_{r}$ = 1.4, and a ShieldIt conductive textile, with thickness of 0.17 mm (total dimensions of 60 × 50 × 0.7 mm^3^). The antenna was fed through a SIW transition and GCPW. The proposed antenna achieved an impedance BW of 7–28 GHz, maximum directive gain of 10.5 dBi, and maximum radiation efficiency of 96%. After investigating the antenna’s performance in free space, its radiation characteristics are examined in a new media as breast to detect a tumour in different considerations such as central tumour with and without skin, and multiple tumours (two and three) within the breast. The reconstructed images proved that the antenna can be an acceptable candidate with high performances working on a media like a breast and later as part of a wearable WBAN system for breast cancer monitoring and imaging.

## Figures and Tables

**Table 1:** Antenna parameters dimensions.

**Parameters (mm)**	**Values**	Parameters (mm)	Values	Parameters (mm)	Values
$W_{s}$	50	$W_{1}$	13	$r_{2}$, $t_{2}$	0.5, 3
$L_{s}$	60	$L_{1}$	19.75	$r_{3}$, $t_{3}$	3.35, 2.8
$W_{p}$	29.5	a	1.8	$r_{4}$, $t_{4}$	1.8, 1.4
$L_{p}$	29.5	b	26	$r_{6}$, $t_{6}$	1.4, 0.7
$r_{1}$, $t_{1}$	4.5, 1.5	$r_{5}$,$\;t_{5}$	1.5, 0.75	$r_{7}$	2.76, 1.25

**Table 2:** Specific absorption rate (SAR) values at different frequencies and standards.

$\mathrm{SAR}/f_{r}$	3.8 GHz (1 g, 10 g)	5.8 GHz (1 g, 10 g)	7 GHz (1 g, 10 g)	28 GHz (1 g, 10 g)
Values (W/Kg)	0.25, 0.071	0.7, 0.171	1.29, 0.520	2.04, 0.690

**Table 3:** Proposed antenna’s gain (dBi) and efficiency (%) for simulation (sim) and measurement (meas) (on: on breast and off: free space).

Parameters/*f_r_* (GHz)	Eff (%) Sim. On	Gain (dBi) Sim. On	Eff (%) Sim. off	Gain (dBi) Sim. off	Eff (%) Meas. on	Gain (dBi) Meas. on	Eff (%) Meas. off	Gain (dBi) Meas. off
3.8	81.90	3.10	83.90	3.12	82.90	3.00	82.90	3.01
5.8	84.90	5.65	85.90	5.50	85.50	5.00	85.19	5.15
7	88.50	5.79	88.00	5.89	87.00	5.39	88.50	5.18
15	88.90	7.63	89.90	7.73	88.90	7.43	89.19	7.27
20	94.90	10.2	95.90	10.10	93.90	10.00	94.90	10.01
24	90.50	9.45	91.50	9.50	91.25	9.15	91.15	9.15
28	90.00	9.23	90.50	9.33	89.50	9.13	90.05	9.03

**Table 4:** Proposed antenna’s performance comparison with similar works.

Ref No.	Dimensions (mm)	BW (GHz)	Max Efficiency (%)	Max Gain (dBi)	Feeding Method	Min SAR
[50]	48 × 39	2.7–10.62	-	1.8	CPW	-
[49]	80 × 67	3.7–10.3	<60	4.53	Full GND	0.09
[51]	50 × 45	2.3–16	85	8	CPW	0.1
[52]	54 × 54	2–12	-	-	TL	0.113
[53]	60 × 60	2–15	95	4	TL	-
Proposed	60 × 50	7–28	96	10.5	SIW-GCPW	0.09
